# Regional differences in farmers’ preferences for a native bee conservation policy: The case of farming communities in Northern and Eastern Thailand

**DOI:** 10.1371/journal.pone.0251206

**Published:** 2021-05-06

**Authors:** Manuel Ernesto Narjes, Christian Lippert

**Affiliations:** Department of Production Theory and Resource Economics, Institute of Farm Management, University of Hohenheim, Stuttgart, Germany; Sichuan University, CHINA

## Abstract

Evidence points to past bee-mediated crop pollination deficits in Chanthaburi province, Eastern Thailand. Conversely, no such evidence has yet been reported for Chiang Mai province (Northern Thailand), suggesting that wild pollination is delivered there above the requirements of local orchards. Discrete choice experiments (DCE) were conducted to elicit the preferences of pollinator-dependent orchard farmers with regard to three pollinator conservation measures and their possible effects on of native bee populations in each region. We fitted random parameter logit (RPL) models on the resulting data to capture preference heterogeneity and to obtain willingness to pay (WTP) point estimates. To test our results’ robustness, we also inspected for scale heterogeneity by fitting generalized mixed logit (GMXL) models on the pooled and individual datasets. This yielded WTP space estimates (i.e., directly from WTP distributions) and made possible the comparison of farmers’ preferences for a native bee conservation policy in both regions. The results hint at significant WTP differences for some of the conservation policy attributes between both provinces. Furthermore, unobserved contributions to choice seem to have been more random in Chiang Mai. Our analyses also suggest that farmers who engage in bee-related activities are WTP more for a conservation policy that includes bee husbandry.

## 1. Introduction

### 1.1. Context: Current status of beekeeping and pollination services in Thailand

With the exception of the European honeybee, all other eight honeybee species are native to Southeast Asia [1]. This region is also characterized by its stingless bee (Apidae, Meliponinae) diversity, with 32 species of the genus *Trigona* identified in Thailand to date [2, 3]. Beekeeping in Thailand traditionally consists of attracting wild swarms of the Eastern honeybee (*Apis cerana* F.) to bait-hives (typically an unsophisticated wooden box or hollowed-out trunk), where the colony will reside until a disturbance (e.g., honey harvest) causes them to abscond [4]. It is also a custom in rural Thailand to keep stingless bees (a practice known as meliponiculture), which has gained economic relevance in the past few decades. Farmers collect colonies from several stingless bee species in the forests and place them in artificial hives (of varying degrees of sophistication) on their farms for their pollination services and to harvest their honey [5, 6].

Native bee husbandry has gained popularity in the Eastern Thai provinces of Chanthaburi and Trat, where–some of the most widely cultivated fruit crops are rambutan (*Nephelium lappaceum*), mangosteen (*Garcinia mangostana*), durian (*Durio zibethinus*) and longan (*Dimocarpus longan*)–orchardists started managing stingless bees to compensate for past pollination deficits [6–8]. Moreover, in our 2013 survey in Chanthaburi, some respondents informed us that prior to finding this solution to the pollination deficit, some orchard farmers of the region had experimented with renting *A*. *mellifera* colonies from beekeepers. They preferred keeping stingless bees over honeybees due to their relatively simple maintenance and shorter flight range, which can protect them from possible contact with pesticides from neighboring farms [8]. Compared to the wide foraging range of honeybees (*A*. *mellifera*), typically reaching distances of 5 km [9], stingless bees have a considerably smaller flight radius: e.g. ~600 m for the subgenus *Trigona* [10]. The anecdotes described above corroborate a similar report by Boongird [7] and raise the suspicion that a local pollination crisis might have taken place in this region in the past.

Habitat loss and fragmentation (through, e.g., deforestation), pesticide overuse and pathogens are among the main drivers of pollinator declines worldwide [11–13]. The occurrence of localized pollinator crises in Thailand is thus not unlikely, given the country’s sustained deforestation rates [14] and the four-fold increase in pesticide imports for agricultural applications over the past decade [15–17]. The official promotion of capital-intensive commercial crops within the framework of a national development strategy was one of the main drivers of deforestation in Thailand in the past [18, 19]. More recently, the continuing increase in the price of rubber (*Hevea brasiliensis*) has led to the conversion of forests in the east, northeast and north of the country, as the rubber plantation area has become limited in Southern Thailand [20, 21]. Furthermore, the overuse of pesticides in Thailand may be a result of the preventive (rather than curative) pesticide application strategy that cash-crop farmers have adopted [22, 23].

Currently, the Northern Thai lowlands are extensively cultivated with longan (*Dimocarpus longan*), a Sapindaceae fruit tree that relies heavily on bee-mediated pollination, particularly from *A*. *cerana* and stingless bees [24–26]. Northern Thailand is the leading exporter of longan worldwide (US$274.3 million in fresh fruit in 2013), with approximately 138,500 ha and 206,000 households (47,300 ha and 69,330 households in Chiang Mai province) devoted to producing this fruit in 2013 [27], thus rendering this region’s economy highly dependent on this crop [28–30]. The extensive cultivation of longan in Northern Thailand has also attracted large-scale beekeeping operations, which (February-March) move their ~120,000 *A*. *mellifera* hives to farms around the Chiang Mai-Lamphun valley each season in search of longan nectar foraging sources. They produce ~4,200 metric tons of longan honey per annum, which is highly valued in the Asian markets, where it can be sold at a premium price [31–33].

Thailand is also one of the world’s leading producers of rambutan and durian, the former of which is a close relative of longan that also depends on bee pollination, i.e., predominantly from stingless bees and *A*. *cerana* [6, 34, 35]. Rambutans are mostly consumed domestically (i.e., ~91% of the country’s total production) and partly exported fresh and canned (~US$20.2 million in 2013). The cultivation of this fruit tree employed 116,200 households and 47,900 ha of land in 2013 and is mainly established in Eastern Thailand, with ~45% of the country’s production concentrated in Chanthaburi province [27, 30, 36]. In Chanthaburi, rambutans are commonly intercropped with mangosteens (*Garcinia mangostana*), which are often heavily sprayed with insecticides to comply with the high aesthetic standards (that, e.g., reject fruit presenting any signs of insect damage on its surface) imposed on fruits destined to export markets [37, 38].

Although the accuracy of this information is disputed, honeybees (*Apis*) and stingless bees have also been credited with the pollination of durians, a belief that is commonly shared among durian farmers in Chanthaburi [7]. In truth, durians owe this service exclusively to nocturnal pollinators [39]. For this reason, a decline in the population of bees would have no consequence on durian yields. Nevertheless, we mention durian in this context as in our study we focus on farmers’ *perceptions* regarding the pollination of their crops.

### 1.2. A policy to conserve native bees in Thailand

Considering the economic importance of the pollination services provided by native bees to Thailand’s orchards, it would make sense to implement a policy to conserve them and their habitats. This is especially relevant for regions such as Northern and Eastern Thailand, where the agricultural output is vulnerable to future pollination shortages.

The International Pollinator Initiative’s Plan of Action (IPI-POA) provides guidelines to enhance wild pollinator conservation and habitat restoration. Its *adaptive management* pillar recommends, among others, the following conservation strategies: *i)* offering farmers bee-friendly alternatives to conventional pesticides (e.g., biological control and integrated pest management); *ii)* encouraging the protection and improvement of natural bee habitats within agro-forest ecosystems; and *iii)* fostering the husbandry of native bee species [40]. We conducted expert interviews and focus group discussions with farmers where we identified these measures as potentially having the greatest impact and implementation chances in Thailand’s current agricultural and political context.

In a recent study, Narjes and Lippert [41] conducted a discrete choice experiment (DCE) with longan farmers in Chiang Mai province. Their per capita WTP for the combined implementation of the native bee conservation measures mentioned above and for avoiding a potential 50% native bee population decline were estimated at €18.1 and €40.5, respectively. These estimates strongly contrast with the comparatively high economic losses from a potential pollination deficit in longan orchards, as approximated using the pollination-dependence ratios given by Blanche et al. [25] and Pham [26]. On the other hand, the estimated farmers’ aggregated WTP exceeds the relatively low investment that implementing such conservation policy would actually cost. The DCE approach thus informs policy makers about the relative support that each conservation strategy demands given the preferences of pollination-dependent crop farmers, who are ultimately the most directly concerned stakeholders.

Besides assessing farmers’ preferences for measures to conserve local native bee populations, we attempt at eliciting the existence value of native bees and the option value of preserving the pollination services they provide (among other value components), both of which are neglected by market prices. Indeed, this study does not attempt at estimating the market value of crop pollination by bees, nor does it offer a detailed examination of markets for pollination services. After all, the arguments for the preservation of bee diversity should reach beyond the crop pollination services that a set of dominant bee species may provide [42].

### 1.3. Recent criticism to the random parameter logit (RPL) model

Narjes and Lippert [41] fitted a random parameter logit (RPL) model that also allowed determining significant dispersions around the mean preferences, which they further explained with idiosyncratic factors such as the respondents’ gender and attitude towards native bees.

However, the RPL model has recently come under criticism for neglecting the fact that choice behavior may be more random for some respondents than for others (i.e., respondent-specific heteroscedastic errors). In other words, the heterogeneity in the preference for a single conservation policy attribute may actually (or partly) result from a scale effect, i.e., all attribute weights are scaled up or down proportionately across individuals [43, 44]. Ignoring such a source of variation (i.e., confounding heteroscedasticity with preference heterogeneity) may result in biased estimates and thus lead to erroneous interpretation and policy conclusions [45]. The RPL model indeed accommodates heterogeneous scaling when all parameters are specified to be random and their corresponding errors are allowed to correlate [46]. Nevertheless, a common practice to obtain WTP estimates in the RPL context is to estimate a non-random cost coefficient (cf. Eq 3 below). With the strong assumption of a homogeneous cost parameter, the researcher is implicitly assuming homogeneous scaling over the population; this would lead to biased estimates (i.e., from confounding scale and preference heterogeneity) in the likely case that either the true scale or cost parameters were indeed random [46].

Fiebig et al. [44] propose tackling this issue with the generalized mixed logit (GMXL) model, which explicitly specifies a scale parameter and thereby can disentangle the sources of preference heterogeneity into randomness in the attribute coefficients and randomness in the overall scale of utility.

### 1.4. The Generalized Mixed Logit (GMXL) model

To comprehend the scale parameter, one has to first formulate an individual farmer’s behavioral choice rule from the researcher’s perspective. The researcher only controls *V*_*ij*_, the “representative” portion of the indirect utility $U_{ij}^{*}$ that a farmer *i* derives from a conservation policy alternative *j* ($U_{ij}^{*},j=1,\ldots ,J$), while the unobserved random “residual” term $\varepsilon_{ij}^{*}$ remains exclusively known to the farmer [47, 48]. Assuming that *V*_*ij*_ is linear additive in the conservation policy attributes *X*_*j*_ and their corresponding taste-weights *β* (henceforth referred to as part-worths), as given by $U_{ij}^{*}=V_{ij}(X_{j})+\varepsilon_{ij}^{*}=\beta \prime X_{j}+\varepsilon_{ij}^{*}$, a utility maximizing farmer would choose the alternative *h* with superior utility from a given set of *J* conservation policy alternatives. Modeling this choice decision requires knowing the density of the unobserved residuals $f(\varepsilon_{ij}^{*})$, which for the *standard logit* model are assumed to be distributed independently and identically (IID), following an extreme value type 1 (EV1) distribution that exhibits $var(\varepsilon_{ij}^{*})=\sigma^{2}(\pi^{2}/6)$. Thereby, the parameter *σ* becomes a scale parameter of the underlying standard EV1 distribution and is therefore often referred to as the scale of utility in the choice analysis literature. Normalizing the residual variance to that of a known theoretical distribution requires a standardization of the utility expression, i.e., moving the unknown scale factor to divide the representative utility *V*_*ij*_. The standardized utility

$$U_{ij}=V_{ij}/\sigma +\varepsilon_{ij}=(\beta^{\prime }/\sigma )X_{j}+\varepsilon_{ij}$$ (1)

therefore results from $U_{ij}=U_{ij}^{*}/\sigma$ and has $var(\varepsilon_{ij}^{*}/\sigma )=var(\varepsilon_{ij})=\pi^{2}/6$. From this transformation, the likelihood of the choice outcome described above can be expressed as the *standard* logit choice probability:

$$P_{ih}=\text{exp}(V_{ih}/\sigma )/\sum_{j=1}^{J}\text{exp}(V_{ij}/\sigma ).$$ (2)

The variance of the unobserved residuals is definitionally linked to the implicit scale of utility. In fact, the standard logit model is usually modeled in its scaled form, resulting in part-worth estimates β* = *β*/*σ* that are not separately identifiable from scale [49]. Nonetheless, given the general IID assumption (i.e., *σ* is constant in the population) one can implicitly cancel out scale by dividing the part-worths of any two attributes *k* = 1,…, *K*. Such coefficient ratios are calculated to, e.g., obtain marginal WTP estimates, as

$$WTP_{k}=-\frac{\beta_{k}/\sigma }{\beta_{c}/\sigma }=-\frac{\beta_{k}}{\beta_{c}},$$ (3)

where *β*_*c*_ weights the attribute related to the costs of implementing the conservation policy.

The unobserved utility variance can be accounted for by *explicitly* parameterizing the standardization step described above as the factor *λ* = 1/*σ* that scales the vector of part-worths, i.e., *U*_*ij*_ = *λV*_*ij*_ + *ε*_*ij*_. Thereby, it also becomes apparent that, for *standard logit*, the scale parameter *λ* equals unity [44, 49, 50]. Intuitively, *λ* is the weight that the respondents (equally) place on the utility they derive from *V*_*ij*_, relative to the residual utility they derive from unobserved factors that contributed to their choice. In other words, the larger the parameter *λ*, the smaller *must* be $var(\varepsilon_{ij}^{*})$.

In reality, the residual variance may differ for different decision makers, i.e., $var(\varepsilon_{ij}^{*})={\sigma_{i}}^{2}(\pi^{2}/6)$, resulting in a heteroskedastic scale that is strictly inversely proportional to the standard deviation of the residuals $\varepsilon_{ij}^{*}$. The *GMXL* specification handles the complexity that arises when simultaneously contemplating heterogeneity in scale and in the part-worths [44]. It maintains the IID assumption for the residual term *ε*_*ij*_, yet assigns part of the unobserved heterogeneity to the scale and part to the part-worths (respectively subscripted to each individual as *λ*_*i*_ and *β*_*i*_), as follows:

$$\beta_{i}=\lambda_{i}[\beta +\Delta z_{i}]+[\gamma +\lambda_{i}(1-\gamma )]\eta_{i},\gamma \in [0,1].$$ (4)

Here *z*_*i*_ are observed individual-specific characteristics (indicating, e.g., whether the respondent believes she has experienced native bee pollination deficits) that induce heterogeneity in the part-worth mean, and *η*_*i*_ embodies *i*-specific (unobserved) deviations from the mean *β* (thus capturing part-worth heterogeneity). In this study, *η*_*i*_ were allowed to induce correlation in the random coefficients *β*_*i*_. Some *β*_*i*_ may only present unobserved heterogeneity (i.e., homogeneous parameter means), in which cases the vector *Δ* is set to zero. The vector *η*_*i*_ can take any distributional form, but in this study it is assumed to be multivariate normal. The extent to which the standard deviation of *η*_*i*_ depends on the scaling of *β* is controlled by the parameter *γ*. As such, setting *γ* = 1 results in GMXL I [*β*_*i*_ = *λ*_*i*_(*β* + *Δz*_*i*_) + *η*_*i*_], a special case of GMXL that assumes that (*β* + *Δz*_*i*_) is independently scaled from the standard deviation of *η*_*i*_, whereas *γ* = 0 [GMXL II: *β*_*i*_ = *λ*_*i*_(*β* + *Δz*_*i*_ + *η*_*i*_)] imposes proportional scaling for both the part-worth means and their variances [44].

Scale heterogeneity, on the other hand, may be expressed as a function of measurable individual-specific variables *g*_*i*_ (indicating, e.g., whether the respondent keeps bees on his farm or not) as follows:

$$\lambda_{i}=exp(\bar{\lambda }+\delta^{\prime }g_{i}+\tau \varepsilon_{0i}),\varepsilon_{0i}\sim N(0,1),$$ (5)

where $\overset{-}{\lambda }$ and *τ* respectively denote a mean parameter and standard deviation of *λ*_*i*_ and *ε*_0*i*_ represents standard normally distributed unobserved heterogeneity. While the exponential form restricts *λ*_*i*_ to being positive, its expected value must be normalized to 1 to identify *β* as the mean vector of utility weights: given that $E[\lambda_{i}]=\text{exp}\left(\overset{-}{\lambda }+\tau^{2}/2\right)$, we set $\overset{-}{\lambda }=-\tau^{2}/2$. This results in scale heterogeneity *λ*_*i*_ that is ~ *LN*(1, *τ*^2^). We thus estimate the parameters that describe the variance of scale and not *λ*_*i*_ itself [44].

The GMXL choice probabilities are conditioned on the unobserved *η*_*i*_ and *ε*_0*i*_. The analytical estimation of such a model would imply solving Eq 2 in a multiple integral that does not have a closed form and therefore must be approximated through computational simulation. Indicating with *y*_*ijt*_ = 1 policy *j* chosen by farmer *i* in choice situation *t*, and with *y*_*ijt*_ = 0 the alternatives not chosen, the simulated probability ${\hat{P}}_{i}$ of observing farmer *i* making a sequence of choices ${\left\{y_{ijt}\right\}}_{t=1}^{T}$ is obtained as follows:

$${\hat{P}}_{i}=\frac{1}{R}\sum_{r=1}^{R}\prod_{t}\prod_{j}{\left(P(j|V(X_{jt}),\varepsilon_{0}^{r},\eta^{r})\right)}^{y_{ijt}}.$$ (6)

Here, the term $P(j{V(X}_{jt}),\varepsilon_{0}^{r},\eta^{r})$ results from inserting (4) and (5) into the logit formula (2) and is solved for *R* random draws $\left\{\varepsilon_{0}^{r},\eta^{r}\right\}$ that are sampled from the distributions underlying *η*_*i*_ and *ε*_0*i*_. This simulation is made iteratively for different population moments of the assumed distributions (i.e., mean and variance-covariance of *β*, collectively denoted as *θ*) and inserted into the log-likelihood function for all *n* farmers. The model estimation consists of finding the parameters *θ* that maximize the simulated log-likelihood function [44, 49].

The GMXL II case offers a framework that handles the challenges resulting from estimating WTP as the ratio of two random part-worths (e.g., the moments of the WTP ratio distribution are undefined). We may first rewrite utility as separable in the monetary attribute *costs*_*j*_ and the *non-monetary* attributes $X_{j}^{nm}:U_{ij}=-\lambda_{i}{\beta_{ic}costs}_{j}+\lambda_{i}\beta_{ik}^{\prime }X_{j}^{nm}+\varepsilon_{ij}$, where $X_{j}^{nm}$ contains all elements of *X*_*j*_ except *x*_1_, the cost attribute, and *k* = 2,…, *K*. Assuming that the cost’s preference heterogeneity is entirely captured by the scale parameter *λ*_*i*_ (i.e., by setting *β*_*ic*_ to one and its corresponding row in *η*_*i*_ to zero), utility can be re-formulated into the following equivalent specification (referred to in the literature as *WTP space*):

$$U_{ij}=-\lambda_{i}[costs_{j}+(1/\beta_{ic})\beta_{ik}^{\prime }X_{j}^{nm}]+\varepsilon_{ij}=-\lambda_{i}[costs_{j}+\phi_{ik}^{\prime }X_{j}^{nm}]+\varepsilon_{ij}.$$ (7)

The random marginal WTP estimates *ϕ*_*ik*_ are directly estimated and independent of scale, while the scaled monetary part-worth $\lambda_{i}=exp(\overset{-}{\lambda }+\delta \prime g_{i}+\tau \varepsilon_{0i})$ is estimated by relaxing the −*τ*^2^/2 restriction on $\overset{-}{\lambda }$ [44, 51–53].

### 1.5. Hypotheses underlying this study

In this study, we contemplate heterogeneity in farmers’ choice behavior regarding alternative interventions to conserve native bees, which should be reflected in part-worth and scale standard deviations that are significantly different from zero. We thus postulate:

*H1*.*1*: There is significant part-worth heterogeneity, i.e., *var*(*η*_*i*_) ≠ 0 (cf. Eq 4) and
*H1*.*2*: There is significant heterogeneity in scale, i.e., *τ* ≠ 0 (cf. Eq 5).

We further hypothesize that such heterogeneity is partly explained by selected idiosyncratic variables that enter the part-worth and scale specifications as the vectors **z**_*i*_ and **g**_*i*_, and therefore we also postulate *H2*: *Δ* ≠ 0 and *H3*: *δ* ≠ 0 as follows:

*H2*.*1*: Farmers who are beekeepers have higher preferences for the conservation of native bees.
*H2*.*2*: Whether founded in evidence or not, the *notion* of a possible pollination deficit attributed by concerned farmers to native bee population declines may also have a positive effect on farmers’ value perceptions.
*H2*.*3*: There are regional differences (i.e., between the two sampled provinces) in the preferences of farmers for the proposed conservation measures.
*H3*.*1*: The presence of scale heterogeneity partly results from a subsample of respondents, who ascribe past pollination deficits to native bee population declines, applying a higher weight on the explained utility *V*_*ij*_ than the others. In other words, we hypothesize that the choice behavior is more consistent among farmers whose memory of past pollination deficits may have imparted them greater preference consensus.
*H3*.*2*: There are differences, between both sampled regions, in the relative importance that farmers place on the unobserved factors that contributed to their choices (i.e., *E*[*λ*_*i*_]_*Chanthaburi*_ ≠ *E*[*λ*_*i*_]_*Chiang Mai*_).

Similarly, scale heterogeneity may be also attributed to:

*H3*.*3*: Gender differences and
*H3*.*4*: A subsample of farmers who allow someone else’s bee hives on their farms and/or are beekeepers themselves. Such direct exposure to honeybees in an agro-ecosystem may sensitize farmers about the importance of conserving native bees and their habitats and/or lead to more informed and thus consistent choices.

Although our analyses address farmers’ perceptions, we would like to stress that the notion of a past pollination deficit attributed to declines in the population of native bees may be more justified for farmers in Chanthaburi than in Chiang Mai: there is scientific and anecdotal evidence indicating that an actual crop-pollination crisis took place in the former (cf. Section 1.1), whereas no such evidence has been reported for the latter. Moreover, we hypothesize that

*H4*: Compared to Chanthaburi farmers, Chiang Mai farmers are more likely to engage in activities involving bees manly for the direct economic benefits from the hive (e.g., honey), rather than to supplement their crops’ pollination.

## 2. Materials and methods

### 2.1. Ethics statement

The research and corresponding surveys presented herein were conducted under approval and abiding by the ethical conduct norms of the National Research Council of Thailand. Moreover, previous to conducting surveys, the community leaders of each of the 16 researched villages were asked for permission. Each interviewed farmer was additionally asked for their consent and offered a compensation that had been agreed upon with the respective community leader.

### 2.2. Experimental design and survey

**Fig 1:**
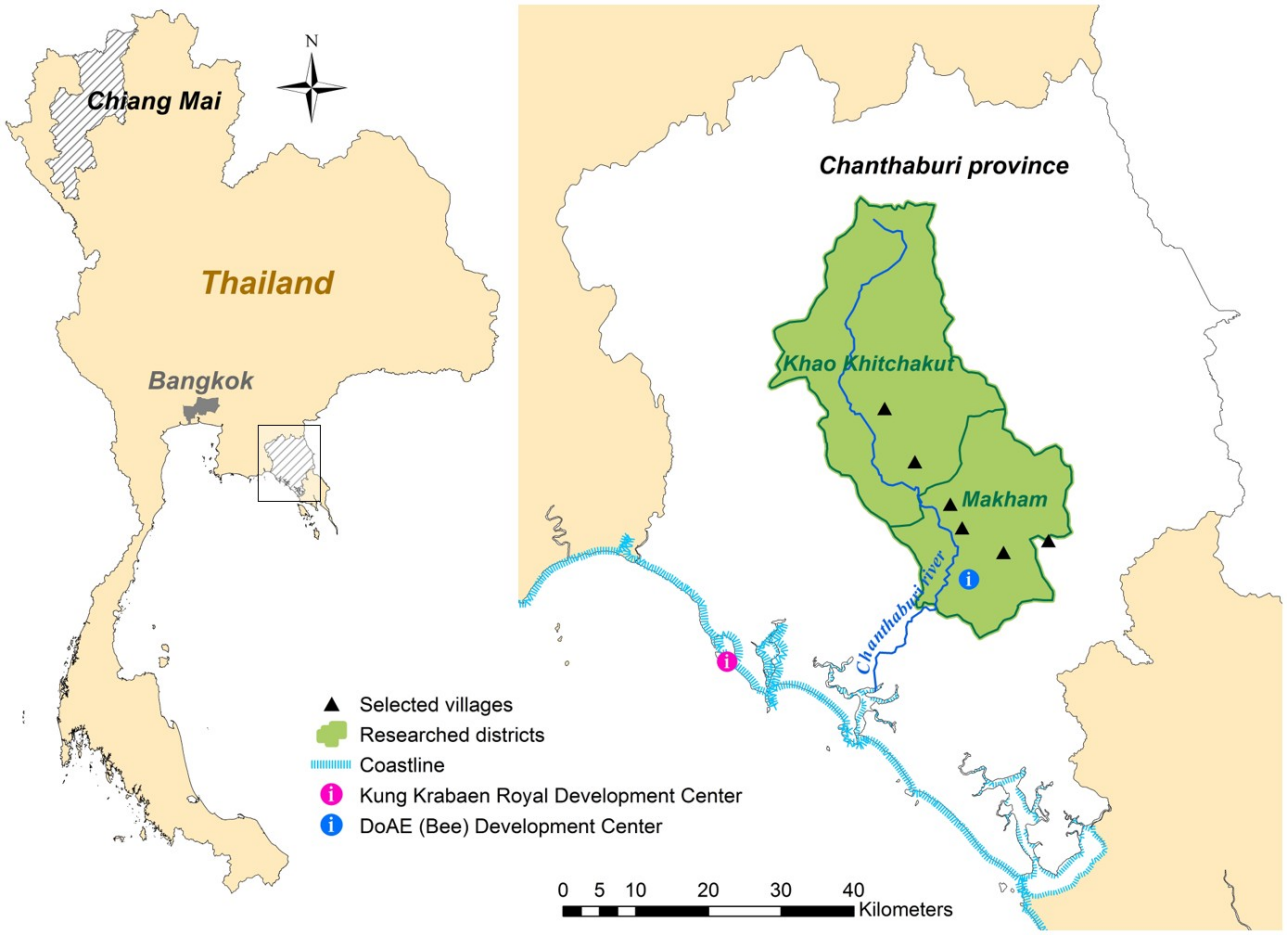
Research area in Chanthaburi. Source: Own representation using vector data from the DCW (Digital Chart of the World) and GADM (Global Administrative Areas) databases made available by DIVA-GIS [55].

This study analyses the datasets resulting from two separate discrete choice experiments (DCEs). The first DCE was conducted with 198 randomly selected individuals from 10 villages in Chiang Mai province (Northern Thailand) in June 2013 [41]. A conditional logit model fitted on the data of the first DCE delivered the prior parameter estimates that were assumed for the efficient design [54] underlying the second DCE. The latter was conducted in November 2013 with 127 farmers in Chanthaburi province (Eastern Thailand). We interviewed 90 respondents in four villages of Makham district, while the remaining 37 were questioned in two villages of Khao Khitchakut province (Fig 1).

In January 2016, the database of the Thai Department of Agricultural Extension [56] registered 3,369 households in Makham, who together farm ~4,380 ha of rambutan, the largest extension of land dedicated to this fruit’s cultivation in a single district of Chanthaburi. The same database registered 1,456 farmers in Khao Khitchakut, who in total cultivate ~1,169 ha of rambutan, rendering this district Chanthaburi’s third largest rambutan producer.

In contrast to the first DCE, a random procedure was not applied to the selection of the Chanthaburi sample. The villages were mandatorily assigned by the provincial administration, and all of the village heads insisted on providing their own selection of respondents. A selection bias may thus affect the representativeness of the results obtained from this dataset.

In twelve different choice occasions, each respondent had to choose one of three alternative scenarios. Each alternative was described by five attributes, namely the three adaptive management techniques we selected from the IPI-POA toolkit, their potential impact on the local population of native bees, and a single advance contribution to cover their combined implementation costs (cf. Section 1.2).

Two of the alternatives, generically named “Policy A” and “Policy B”, varied throughout the twelve choice sets by manifesting different attribute levels (Table 1), thus presenting different hypothetical scenarios of conservation policy implementation. These scenarios were associated with the full range of native bee population changes (i.e., −50, 0, +50) and were contrasted with an unvarying hypothetical status quo alternative, which described the absence of any conservation strategy (i.e., at zero cost) that would lead to a decline of half of the local native bees.

**Table 1:** Choice alternative attributes, corresponding design levels and other variable definitions.

*Definition*	*Levels*	*coding*	*variable name*
*Bee conservation policy attributes (variables appearing in choice sets)*			
Bee-friendly pest control	no ^a)^, yes	dummy	*PEST*
Improving native bee habitat	no ^a)^, yes	dummy	*HAB*
Native bee husbandry	no ^a)^, yes	dummy	*BEEKP*
Changes in native bee population (%)	−50 ^a)^, 0, +50	2 dummies	*NB_DEC* (−0%), *NB_INC* (+50%)
Policy implementation costs (THB) ^b)^	0 ^a)^, 250, 500, 750	continuous	*COSTS*
*Idiosyncratic variables*			
Beekeeper (own bees)		dummy	*BEEKEEPER*
Keeps bees on her farm (own bees or someone else’s)		dummy	*BEE_FARM*
Engages in at least one of the following activities: beekeeping, hunting for wild bee honey or charging a fee to allow someone else’s bees to forage on her farm		dummy	*ECON_BEE*
Rated native bees’ effect on her crop yields as good or excellent		dummy	*POS_BEE*
Believes he has experienced a native bee-pollination shortage		dummy	*POLL_DEC*
Farmer in Chanthaburi		dummy	*CHB*
Male respondent		dummy	*MALE*

^a)^ Attributes fixed at these levels for the status quo alternative.

^b)^ The cost attribute represents a one-time fee that the farming households would pay to the local authorities for the implementation of the chosen policy alternative. €1 = 39.3048 Thai baht (THB), as of June 1, 2013.

### 2.3. Discrete choice experiments

We elicited the preferences of longan (*Dimocarpus longan*) farmers in Chiang Mai province (Northern Thailand) and of rambutan (*Nephelium lappaceum*) farmers in Chanthaburi province (Eastern Thailand) regarding alternative policy scenarios aimed at conserving native pollinating bees. Two discrete choice experiments (DCEs) were conducted in 2013, the resulting data of which was fitted with the random parameter logit (RPL) and the generalized mixed logit (GMXL) models to identify possible sources of heterogeneity in respondents’ willingness to pay (WTP) for three recommended conservation measures (cf. Section 1.2) and for their potential effect on local native bees. The WTP for such measures are expected to be higher among those farmers who are beekeepers, considering that a previous, firsthand exposure to honeybees interacting with an agro-ecosystem could increase the awareness about the importance of conserving their native equivalents and habitat.

We also inspected for choice behavior differences between the two regions and whether or not such differences could be traced back to individuals who *believe* having experienced an insufficient crop pollination due to native bee population declines, as this has been reported for Chanthaburi. In this regard, we emphasize that choice analysis addresses the value of *perceived*, rather than actual, changes in the provision of an environmental good. We also stress that, in the context of this study’s DCE, both the implementation of a conservation policy and its effect on the local native bee population are hypothetical. Accordingly, the preferences of the surveyed farmer population do not have to be consistent with the ecological implications one would expect from actually implementing a conservation policy, as farmers do not have to be aware of ecological processes to form their preferences. Furthermore, we inspect the potential influence of idiosyncratic characteristics on the overall importance that respondents place on the conservation policy attributes that we propose, relative to the unaccounted factors that also influenced their choices.

### 2.4. Model specifications and selection procedure

We used NLOGIT 5/LIMDEP 10 software to examine sources of choice heterogeneity with the RPL and GMXL models. The GMXL I form is assumed in the analyses of this section to induce covariance in the vector of mean part-worths while allowing for independent standard deviations, thereby reducing the confounding of scale and part-worth heterogeneities. For a more detailed discussion of this issue refer to Hess and Rose [57]. We relied on the Bayesian information criterion (For *N* choice observations and *K* parameters, BIC = −2ln *L* + *K* ln *N*, where *L* is the maximized value of the likelihood function) to indicate which model offers a better fit and is thus preferred. Herewith, a lower BIC from an RPL model would suggest that choice heterogeneity is better explained by randomness in the part-worths alone. Conversely, preferring a GMXL could indicate a non-negligible contribution of scale variance to choice heterogeneity. The Akaike information criterion (AIC = −2ln *L* + 2*K*), on the other hand, guided the choice between variations of models that resulted from explaining heterogeneity in the part-worth means with idiosyncratic variables; the large number of parameters that is necessary to capture complex choice behavior with these models (e.g., error correlations) would otherwise be heavily penalized by BIC [44]. For *N* ≥ 8, BIC tends to choose models that are more parsimonious than those favored by AIC, as it imposes a larger penalty on the added parameters [58]. Between two candidate models, a *Δ*BIC > 2 is considered sufficient evidence to choose the model with the lower BIC [59]. Given the few additional GMXL parameters, Fiebig et al. [44] conclude that the BIC is the most reliable criterion to indicate whether scale heterogeneity is present or not. The above described procedure offers a systematic approach to a hypotheses-driven model selection, which is necessary in view of the extremely large number of model specifications one could formulate in order to test the hypotheses postulated in this study.

For our first analysis, we fitted several GMXL model specifications to the Chiang Mai dataset, in order to determine whether scale heterogeneity is present. Models M2 through M5 capture scale and part-worth heterogeneity with different idiosyncratic characteristics, under specifications that minimize BIC and AIC, and are to be compared with the “all-parameters-random” RPL model estimated by Narjes and Lippert [41]. For reference purposes, we additionally fitted model M1, a baseline RPL, with the full parameter vector set to be random and correlated *η*_*i*_, and without interaction terms in the mean part-worths in order to keep it at the minimum necessary number of parameters. Model M2 is a GMXL that captures unexplained scale and part-worths heterogeneity, while Models M3 and M4 partly explain scale heterogeneity with either of two dummies that indicate whether the respondents *i)* believe they experienced a native bee-pollination shortage (POLL_DEC) or *ii)* keep their own or someone else’s bees on their farm (BEE_FARM). Model M5 further explains heterogeneity in the part-worth means of BEEKP and NB_INC; the former with a dummy that indicates whether the farmer is a beekeeper (BEEKEEPER) and the latter with a dummy (ECON_BEE) indicating whether the household engages in at least one of three economic activities involving bees, i.e., beekeeping, hunting for wild bee honey and/or charging other beekeepers a fee to let their colonies forage longan nectar on their farms. This latter model resulted from a stepwise backward elimination derived from the “All-parameters-random” model reported by Narjes and Lippert [41].

We also looked for sources of scale and part-worth heterogeneity in the Chanthaburi dataset and for heteroscedasticity that may result from pooling the Chiang Mai and Chanthaburi datasets. For the latter analysis, we defined a dummy indicative of Chanthaburi respondents (CHB) to capture possible scale effects resulting from differences in the underlying experimental design and/or in the regions.

The analyses described above helped us disentangling the sources of heterogeneity in scale and random part-worths, thus guiding the WTP space specifications with *γ* fixed at 0 (i.e., GMXL II, according to Eq 7) for the models M12 through M15. We first juxtaposed WTP point estimates (M11, obtained from assuming a homogeneous COSTS part-worth in M6) with WTP space estimates (M12, guided by the part-worth and scale treatments of M8) for Chanthaburi, in order to check for consistency in the signs and orders of magnitude. Similarly, we estimated two WTP space models for Chiang Mai in order to compare them with the WTP point estimates model obtained by Narjes and Lippert [41]: the first WTP space model (M13) was specified analogously to its WTP point estimates counterpart, while the second WTP space model (M14) was guided by the specification in M5. Finally, we tested for significant differences in the WTP of respondents from both regions by fitting a WTP space model (M14) on the pooled data, in which CHB explains heterogeneity in the conservation policy part-worths and in scale (the latter to rule out any possible regional effect on heteroscedasticity).

## 3. Results

### 3.1. Descriptive statistics

The descriptive data suggest important differences in the socio-demographics and farming practices of the Chiang Mai and Chanthaburi samples. Table 2 describes and compares these farming communities with selected variables, some of which we used to explain heterogeneity in part-worths and scale.

**Table 2:** Sample characteristics based on respondents’ per-household values, 2013.

*Variable*	*Chiang Mai* ^a)^		*Chanthaburi* ^b)^	
	*Mean*	*(SD)*	*Mean*	*(SD)*
Age (years)	55.76	(11.98)	44.89*** ^g)^	(12.36)
Cultivated acreage (rai) ^c)^				
Longan	5.92	(6.09)	7.47	(5.82)
Rambutan	-		9.54	(9.97)
Durian	-		9.31	(9.65)
Total cultivated acreage (rai) ^c)^	7.15	(7.49)	25.42*** ^g)^	(21.10)
Net annual agricultural income (THB) ^d)^	76,415	(96,822)	334,543*** ^g)^	(361,809)
Net total annual income (THB) ^d)^	255,005	(654,567)	362,861	(367,551)
	*Variable name*	*Sample shares (%)*		
Male	MALE	58.59	49.61	
Main occupation: self-employed in agriculture	-	85.35	99.21*** ^h)^	
Longan farmers ^i)^ [Total cultivated area (rai)]	-	100.00 [1172.7]	13.39 [127.0]	
Rambutan farmers ^i)^ [Total cultivated area (rai)]	-	0.00 [0.0]	70.87 [859.0]	
Durian farmers ^i)^ [Total cultivated area (rai)]	-	0.00 [0.0]	88.19 [1043.2]	
Keep bees on their farm ^e)^	BEE_FARM	38.89	62.99*** ^h)^	
Their own	BEEKEEPER	15.66	59.84*** ^h)^	
Someone else’s	-	28.79	9.45*** ^h)^	
Honey hunters ^f)^	-	20.71	29.13	
Engage in at least one of the above bee-related activities	ECON_BEE	50.00	72.44*** ^h)^	
Completed only six years of primary school [no formal education]	-	77.27	57.48*** ^h)^	
	-	[5.56]	[1.57]	
Rated native bees’ effect on their crop yields as good or excellent	POS_BEE	87.37	96.06** ^h)^	
Self-assessed knowledge regarding pollination before the survey: rated at least basic or [high]	-	90.40	94.49	
	-	[7.58]	[24.41]*** ^h)^	
Blame past yield declines on bee pollination deficits	POLL_DEC	38.38	47.24	

^a)^ *n =* 198 respondents.

^b)^ *n =* 127 respondents.

^c)^ 1 rai *=* 0.16 ha.

^d)^ €1 = 39.3048 Thai baht (THB), as of June 1, 2013.

^e)^ *A*. *mellifera* or native bees (i.e., *A*. *cerana* and/or stingless bee spp.).

^f)^ Harvesting honey from wild bees in the forest. Significantly different from Chiang Mai sample with *p<0.05, ** *p*<0.01 and *** *p*<0.001.

^g)^ Student’s t-test.

^h)^ Chi-squared test of independence.

^i)^ Cultivates at least (not exclusively) 1 ngan (0.25 rai) of specified crop. Source: own calculation.

It becomes evident that agriculture plays a more important economic role among the surveyed households of Chanthaburi than it does in the longan farming population of Chiang Mai: with a 92% share derived from agriculture, Chanthaburi farmers earn 42% higher net incomes than the latter. Household incomes of Chiang Mai longan farms, on the other hand, consist mainly in off-farm earnings, i.e., 70% of net income.

Notably, the function of native bees as crop pollinators (POS_BEE) was more emphatically acknowledged by the respondents in Chanthaburi, who also present a significantly higher engagement in beekeeping activities (BEEKEEPER and BEE_FARM) than longan farmers in Chiang Mai. Moreover, being a Chanthaburi beekeeper is significantly related to POLL_DEC, the notion of a past crop pollination deficit that the farmer attributes to local native bee declines [*χ*^2^(1) = 4.88, *p* = 0.027; tetrachoric correlation: *ρ* = 0.308, *SE* = 0.132, *p*<0.05]. Such association could not be determined for the Chiang Mai sample, where beekeeping is thus more likely to be practiced only for its direct benefits.

Also worth mentioning is the significant relationship between education and self-assessed knowledge regarding pollination [*χ*^2^(10) = 21.62, *p*<0.05] in the pooled dataset, where 171 respondents fell into the category of having acquired primary school education and having rated their pollination knowledge as basic. The independence of these categories could nevertheless not be rejected within the separate datasets, suggesting that their significant association in the pooled data results from a regional effect that may be explained with, e.g., the higher education level and higher pollination awareness in Chanthaburi. In fact, several initiatives supporting the research and development of native beekeeping have been hosted in Chanthaburi, including the Provincial Agricultural Occupation Promotion and Development Center (Bee) of the DoAE, the Royal Development Study Center in Kung Krabaen bay (Fig 1) and a project sponsored by H.R.H. Princess Maha Chakri Sirindhorn in Makham district that trained local farmers on how to produce wooden stingless bee hive boxes [6, 60–62].

### 3.2. Identifying sources of preference and overall scale heterogeneity

Table 3 reports the RPL and GMXL models we fitted to the Chiang Mai dataset for our first analysis, in which we aim at determining sources of heterogeneity in scale and in the random part-worths.

**Table 3:** Random parameter logit and generalized mixed logit (*γ* fixed at 1) models fitted on Chiang Mai dataset.

*Variable*	*M1*: *RPL*		*M2*: *GMXL*		*M3*: *GMXL*		*M4*: *GMXL*		*M5*: *GMXL*	
	*Mean* ^a)^	*SD*	*Mean* ^a)^	*SD*	*Mean* ^a)^	*SD*	*Mean* ^a)^	*SD*	*Mean* ^a)^	*SD*
PEST	1.29287***	1.48343***	1.56450***	1.79170***	1.56653***	1.80074***	1.55832***	1.66477***	1.45318***	1.41726***
HAB	1.28213***	0.55993*	1.45823***	1.35947***	1.46612***	1.38437***	1.50250***	1.03063***	1.47212***	0.39712*
BEEKP	0.84121***	1.31470***	1.02148***	1.67182***	1.03325***	1.66406***	1.03743***	1.68863***	0.85419***	1.61024***
NB_DEC	−5.23836***	2.73449***	−7.30680***	1.89658***	−7.34405***	1.84724***	−7.02944***	1.80021***	−6.39451***	1.65209***
NB_INC	3.26981***	2.01934***	4.19878***	2.58636***	4.24664***	2.54772***	4.19584***	2.14896***	3.39856***	1.85950***
COSTS (THB)	−0.00448***	0.00320***	−0.00582***	0.00383***	−0.00581***	0.00388***	−0.00569***	0.00329***	−0.00503***	0.00383***
*Heterogeneity in random parameter mean (*Δ*)*										
BEEKEEPER (*z*_*i*_): BEEKP	-		-		-		-		1.03810**	
ECON_BEE (*z*_*i*_): NB_INC	-		-		-		-		1.42583**	
*Parameters in scale*: (*τ*)	-		0.70201***		0.75972***		0.73007***		0.64482***	
BEE_FARM (*δ*)	-		-		−0.22921***		-		-	
POLL_DEC (*δ*)	-		-		-		−0.40490***		−0.38335***	
Log-Likelihood (LL) ^b)^	−1470.4834		−1462.9989		−1462.1765		−1454.0544		−1446.7320	
Parameters (*K*)	27		28		29		29		31	
BIC/*N*; [AIC/*N*] ^b)^	1.326[1.261]		1.323[1.255]		1.326[1.255]		1.319[1.248]		1.319[1.244]	
Adjusted [McFadden] R^2^ ^d)^	0.353[0.357]		0.356[0.360]		0.356[0.360]		0.360[0.364]		0.363[0.367]	
LRT ^c)^ ^d)^ (*df*) *χ*^2^	(25) 1629.4328***		(26) 1644.4018***		(27) 1646.0464***		(27) 1662.2907***		(29) 1676.9355***	

Refer to Table 1 for variable definitions. *N =* 2376 choice observations from 198 respondents.

^a)^ Significance levels: * *p* < 0.05, ** *p* < 0.01, *** *p* < 0.001.

^b)^ Compare to “all-parameters-random model” by Narjes and Lippert [41]: *K* = 32, LL = −1455.6685, BIC/N = 1.330, AIC/N = 1.252.

^c)^ Likelihood ratio test.

^d)^ Based on the LL function of a restricted model with two intercepts only, i.e., choice probabilities set at each alternative’s sample shares. Source: own calculation.

According to the obtained BICs, models M2, M4 and M5 are superior to M1 and to the RPL model by Narjes and Lippert [41], i.e., a significant amount of heterogeneity can be attributed to scale differences. Furthermore, the negative coefficient in POLL_DEC (M4 and M5) suggests a reduced scale (corresponding to a higher *σ*) among those farmers who stated that they had experienced a pollination deficit, which can be ascribed to the relatively higher contribution of unobserved factors to the utility they realized from their choices, i.e., a larger variance in residuals. Moreover, a reduced scale could also be significantly traced back to farmers keeping bees on their farms (M3) and to farmers’ increasing age [model not reported: *τ* = 0.95487 (*p*<0.001) and *δ* = −0.00613 (*p*<0.01); *χ*^2^(27) = 1645.20, *p* = 0.00; BIC/N = 1.326; AIC/N = 1.256]. No important BIC difference between models M4 and M5 was found, yet AIC prefers the latter, indicating a model fit improvement from adding interaction terms that explain the means of BEEKP and NB_INC.

Table 4 reports the RPL and GMXL models fitted to the Chanthaburi dataset. Model M7 hints at a significantly (yet not very) heterogeneous scale that a further model (M8) explained with a dummy indicating gender (MALE), according to which male respondents would have made fewer random choices. These results notwithstanding, M6 (the baseline RPL model) was preferred by BIC (yet not by AIC), signaling that the heterogeneous choice behavior in Chanthaburi is better explained by solely capturing it with the random part-worths. Table 4 also reports the GMXL model (M9) that was fitted on the pooled datasets (i.e., Chiang Mai + Chanthaburi). A further model, where CHB was specified to capture heterogeneity in both scale and the part-worth means, resulted in a non-significant effect of CHB on scale [model not reported in this paper’s results tables: *τ* = 0.56502 (*p*<0.001) and *δ* = −0.02011 (*p* = 0.79); *χ*^2^(30) = 2438.00, *p* = 0.00; BIC/N = 1.339; AIC/N = 1.288]. We thus compared M9 to a model in which CHB only has an effect on the part-worth means of the conservation policy attributes (M10), resulting in the latter being preferred by BIC and AIC in spite of its greater number of parameters.

**Table 4:** Random parameter logit (RPL) and generalized mixed logit (GMXL; *γ* fixed at 1) models fitted on Chanthaburi dataset and GMXL fitted on pooled data.

*Variable*	*M6*: *RPL Chanthaburi* ^d)^		*M7*: *GMXL Chanthaburi* ^d)^		*M8*: *GMXL Chanthaburi* ^d)^		*M9*: *GMXL pooled data* ^e)^		*M10*: *GMXL pooled data* ^e)^	
	*Mean* ^a)^	*SD*	*Mean* ^a)^	*SD*	*Mean* ^a)^	*SD*	*Mean* ^a)^	*SD*	*Mean* ^a)^	*SD*
PEST	1.21990***	1.65001***	1.06637***	1.54174***	1.07457***	1.55720***	1.19755***	1.52034***	1.17471***	1.58054***
HAB	1.74206***	1.49563***	1.69797***	1.24242***	1.63901***	1.21620***	1.25031***	1.06475***	1.16678***	1.04974***
BEEKP	1.04907***	1.35912***	0.98355***	1.31533***	0.95039***	1.25279***	0.84325***	1.43884***	0.72991***	1.39975***
NB_DEC	−2.66051***	1.42451***	−3.02800***	1.73858***	−2.89768***	0.90328***	−5.47557***	1.47275***	−5.97960***	1.42407***
NB_INC	2.04788***	1.49773***	2.17420***	1.63267***	2.30901***	1.48040***	3.30631***	1.35985***	3.35288***	1.72152***
COSTS (THB)	−0.00232***	0.00238***	−0.00241***	0.00260***	−0.00253***	0.00235***	−0.00413***	0.00291***	−0.00411***	0.00311***
*Heterogeneity in random parameter mean (Δ)*										
CHB (*z*_*i*_): HAB	-		-		-		-		1.09402***	
CHB (*z*_*i*_): BEEKP	-		-		-		-		0.79023***	
CHB (*z*_*i*_): NB_DEC	-		-		-		-		2.46479***	
*Parameters in scale*: (*τ)*	-		0.15063***		0.23764***		0.53793***		0.56236***	
MALE (*δ*)	-		-		0.52483***		-		-	
CHB (*δ*)	-		-		-		0.23674***		-	
Log-Likelihood (LL)	−995.5278		−996.2910		−993.1677		−2488.6325		−2478.7133	
Parameters (*K*)	27		28		29		29		31	
BIC/*N*; [AIC/*N*] ^d)^ ^e)^	1.436[1.342]		1.442[1.344]		1.443[1.341]		1.338[1.291]		1.337[1.287]	
Adjusted [McFadden] R^2^ ^c)^	0.286[0.293]		0.286[0.292]		0.288[0.294]		0.327[0.325]		0.327[0.330]	
LRT ^b)^ ^c)^ (*df*) *χ*^2^	(25) 823.4394***		(26) 821.9130***		(27) 828.1596***		(27) 2418.1554***		(29) 2437.9939***	

Refer to Table 1 for variable definitions.

^a)^ Significance levels: * *p* < 0.05, ** *p* < 0.01, *** *p* < 0.001.

^b)^ Likelihood ratio test.

^c)^ Based on the LL function of a restricted model with two intercepts only, i.e., choice probabilities set at each alternative’s sample shares.

^d)^ Chanthaburi dataset: *N =* 1524 choice observations from 127 respondents.

^e)^ Pooled data (Chiang Mai + Chanthaburi): *N =* 3900 choice observations from 325 respondents. Source: own calculation.

We also tested whether POLL_DEC can capture part of the part-worth heterogeneity in the Chanthaburi dataset, an effect that for Chiang Mai had already been discarded following a stepwise approach by Narjes and Lippert [41]. A positive yet statistically (not quite) significant effect was detected for the POLL_DEC×BEEKP interaction [model not reported in this paper’s results tables: *β* = 0.798 (*p*<0.01) and Δ = 0.484 (*p* = 0.057); *K* = 28; *χ*^2^(26) = 827.00, *p* = 0.00; BIC/N = 1.439; AIC/N = 1.341]. This result suggests that those farmers in Chanthaburi, who believe having experienced a bee-mediated pollination deficit, value the beekeeping measure more than the others.

### 3.3. Willingness to pay estimation

Table 5 reports WTP estimates for Chanthaburi and Chiang Mai, modelled separately, and for both regions modelled together. The estimates from M11 (i.e., WTP point estimates) and M12 (i.e., WTP space estimates) seem fairly robust, although PEST and BEEKP were 21% and 10.4% lower in M12, respectively, which would thus render the WTP space estimates comparatively more conservative. Nevertheless, M11 was preferred by both information criteria, and the non-significant *τ* in M12 corroborates a negligible scale heterogeneity in the Chanthaburi dataset. A further model (with lower explanatory performance) suggested that, as in the case of Chiang Mai, Chanthaburi beekeepers have a higher preference for BEEKP than non-beekeepers, the mean WTP of the latter being not significantly different from zero [model not reported: Δ*WTP*_*BEEKP*_ = THB359, *p*<0.01; *K* = 22; *χ*^2^(20) = 785.73, *p* = 0.00; BIC/N = 1.437; AIC/N = 1.360]. It is thus not surprising that the model without the BEEKEEPER×BEEKP term is preferred: the majority of respondents in Chanthaburi are beekeepers and therefore BEEKP suffices to capture the mean preference for the whole sample. Similarly, in another model, Chanthaburi farmers who present the POLL_DEC characteristic (regardless of being beekeepers or not) are WTP more than those who did not believe having experienced a native bee-pollination shortage [model not reported: ${\bar{WTP}}_{BEEKP}=THB251$ (*p* = 0.054) and Δ*WTP*_*BEEKP*_ = THB237 (*p* = 0.07); *K* = 22; *χ*^2^(20) = 782.13, *p* = 0.00; BIC/N = 1.439; AIC/N = 1.362].

**Table 5:** Willingness to pay (WTP) estimates in Thai Baht (THB) for Chanthaburi, Chiang Mai and pooled datasets (costs parameter fixed at 1 with std. dev = 0).

*Variable*	*M11*: *Chanthaburi*		*M12*: *Chanthaburi*		*M13*: *Chiang Mai* ^g)^		*M14*: *Chiang Mai* ^g)^		*M15*: *pooled data*	
	*WTP* ^a)^ ^d)^ ^e)^	*SD*	*WTP* ^a)^ ^d)^ ^f)^	*SD*	*WTP* ^a)^ ^d)^ ^f)^	*SD*	*WTP* ^a)^ ^d)^ ^f)^	*SD*	*WTP* ^a)^ ^d)^ ^f)^	*SD*
PEST	529.865***	715.175***	419.899***	711.365***	133.412	508.534***	290.413***	577.934***	348.093***	512.506***
HAB	793.349***	988.039***	775.677***	969.279***	154.559	333.378***	308.232***	407.100***	355.655***	450.952***
BEEKP	461.883***	903.766***	413.929***	794.669***	177.437***	318.904***	163.212***	357.238***	216.880***	452.149***
NB_DEC	−1380.110***	1017.920***	−1295.380***	581.134**	−1247.970***	515.354***	−1445.340***	548.637***	−1305.450***	629.691***
NB_INC	939.196***	704.132***	1004.630***	752.330***	687.127***	370.411***	705.825***	474.329***	898.402***	576.426***
*Heterogeneity in random parameter mean (ΔWTP)*										
BEEKEEPER (*z*_*i*_): BEEKP	-		-		186.413*		211.108**		-	
ECON_BEE (*z*_*i*_): NB_INC	-		-		225.422***		220.832*		-	
POS_BEE (*z*_*i*_): PEST	-		-		204.683		-		-	
POS_BEE (*z*_*i*_): HAB	-		-		178.478*		-		-	
CHB (*z*_*i*_): HAB	-		-		-		-		356.052***	
CHB (*z*_*i*_): BEEKP	-		-		-		-		265.419***	
CHB (*z*_*i*_): NB_DEC	-		-		-		-		350.696**	
*Parameters in scale*: (*τ)*	-		0.25310		0.75980***		0.61819***		0.54113***	
MALE (*δ*)	-		0.66499		-		-		-	
POLL_DEC (*δ*)	-		-		−0.29629		−15.55560		-	
CHB (*δ*)	-		-		-		-		0.32619	
Log-Likelihood (LL)	−1012.8470		−1015.5205		−1500.6317		−1495.6878		−2558.9053	
Parameters (*K*); Obs. [*N*]	21[1524]		23[1524]		27[2376]		25[2376]		26[3900]	
BIC/*N*; [AIC/*N*]	1.430[1.357]		1.443[1.363]		1.351[1.286]		1.341[1.280]		1.367[1.326]	
Adjusted [McFadden] R^2^ ^c)^	0.275[0.280]		0.273[0.278]		0.340[0.343]		0.342[0.346]		0.306[0.308]	
LRT ^b)^ ^c)^ (*df*) *χ*^2^	(19) 788.8001***		(21) 783.4539***		(25) 1569.1360***		(23) 1579.0240***		(24) 2277.6098***	

Refer to Table 1 for variable definitions.

^a)^ Significance levels: * *p* < 0.05, ** *p* < 0.01, *** *p* < 0.001.

^b)^ Likelihood ratio test.

^c)^ Based on the LL function of a restricted model with two intercepts only, i.e., choice probabilities set at each alternative’s sample shares.

^d)^ €1 = 39.3048 Thai baht (THB), as of June 1, 2013.

^e)^ WTP point estimates from RPL with fixed costs (M6, Table 4).

^f)^ WTP space (*γ* fixed at 0) models.

^g)^ Compare to “fixed-costs model” by Narjes and Lippert [41]: *K* = 25, LL = −1513.8718, BIC/N = 1.356, AIC/N = 1.295. Source: own calculation.

Model M13 also yielded estimates slightly similar to those reported by Narjes and Lippert (2016), insofar as the mean WTP for PEST and HAB was not significantly different from zero. Additionally, the mean WTP for NB_INC and BEEKP, and the estimates for BEEKEEPER×BEEKP and ECON_BEE×NB_INC, were almost identical. The remaining estimates, nevertheless, differed greatly between the two models. On the other hand, M14 is preferred over both M13 and the point estimates model offered by Narjes and Lippert [41] according to both information criteria. Moreover, the significant estimates for both of M14’s interaction terms give further evidence of these terms’ robustness.

The final comparison hints at significantly different preferences for conservation policy attributes between respondents of the two sampled locations. According to M15, Chiang Mai farmers were willing to pay ~THB356 for the implementation of the native bee habitat measure, while Chanthaburi farmers were WTP an *additional* THB356. This difference is slightly smaller than the ~THB485 difference between the estimates corresponding to M11 and M14. On the other hand, the additional THB265 that, according to M15, Chanthaburi farmers are WTP for BEEKP is a result almost identical to the corresponding difference between M11 and M14, given that Chiang Mai farmers are willing to pay THB196 for BEEKP in average (weighted by the 0.16 share of those keeping their own bees, presented in Table 2).

With regard to the remaining attributes (with the exception of NB_DEC), the estimates of M15 also resemble the differences between the estimates from M11 and M14, provided that the weights corresponding to the sample size and interaction term shares are correctly applied. These results also hint at the robustness of the WTP estimates from the selected models.

The idiosyncratic effects that were significant on the scale (i.e., *δ*) of the GMXL I models were not significant in all of the analogous WTP space (i.e., GMXL II) models. This may result from the fact that in GMXL II, *λ*_*i*_ not only weights the means of the random parameters (as in GMXL I), but also weights in equal proportion their corresponding standard deviations (cf. Eq 4).

## 4. Discussion

We could not reject *H1*.*1* as there was significant part-worth heterogeneity for all attributes in all reported models. The sources of farmers’ choice behavior heterogeneity are an important issue in this article, and thus it should be recalled that scale heteroscedasticity was especially relevant in the Chiang Mai dataset. The negative sign in the POLL_DEC coefficient (i.e., farmers who believe they experienced a bee-mediated crop pollination decline, place a relatively higher weight on unobserved attributes when making their choices) nevertheless came up as a surprise: we expected these farmers to place a higher weight on conservation policy attributes. We thus keep *H3*.*1* with an unanticipated negative sign. A possible interpretation of this counterintuitive result is that an important pollinator deficit has not yet been experienced (or perceived as such) among farmers in Chiang Mai and that POLL_DEC instead captured the random answers of respondents who did not fully understand the ecosystem service provided by the bees. In other words, farmers with a low understanding of and/or skepticism about the need for an intervention to conserve native pollinators may have introduced different subjective factors into their choices, thus contributing to an increased variability in the residuals; such farmers are also prone to misunderstanding the question captured by the dummy POLL_DEC, to which they may have randomly answered yes or no. Explaining scale differences may therefore point at a heterogeneous level of relevant knowledge (with respect to the importance of conserving the environmental good in question) or understanding of the DCE exercise on the part of the respondents. It may nevertheless also indicate that some attributes that were relevant to the choice decisions of a group of respondents were ignored by the researcher.

The supposition of a poor understanding of pollination services in Chiang Mai, relative to Chanthaburi, is supported by the descriptive statistics: the latter presents a significantly larger share of individuals that acknowledge the positive effect of native bees to crop pollination and with significantly higher self-assessed knowledge regarding this ecosystem service (Table 2). Although not directly providing evidence to test *H4*, a further indication of a lower awareness for the importance of bee-mediated pollination in Chiang Mai is given by the fact that there is a higher engagement in beekeeping in Chanthaburi, which only in this province correlates with the notion of having experienced bee-mediated pollination shortages. Between the two provinces, Chiang Mai farmers are thus more likely to perceive hive products as the only benefits they derive from bees.

Accounting for scale heterogeneity in the Chiang Mai dataset indeed resulted in a simpler model (with respect to the interactions in the part-worth means), suggesting that the models offered by Narjes and Lippert [41] were over-parameterized in the part-worth vector and may have yielded slightly biased estimates. Farmers in Chanthaburi, on the other hand, seemed consistent in their choices (i.e., they placed a comparatively homogeneous weight on the policy attributes relative to the residual utility contributions), a behavior that could be attributed to a better understanding of the importance of native bees for the pollination of their crops. This reasoning is not farfetched considering the local evidence from *actual* past pollination deficits in Chanthaburi and the efforts that have been summoned to counteract such problems in this region.

The positive effect that CHB has on scale when modeling the pooled data (M9) may lead to the conclusion that Chanthaburi farmers place a greater weight than Chiang Mai farmers do on the overall conservation policy relative to *ε*_*ij*_. This regional effect, nevertheless, is more dominant as an interaction term on the individual attributes (M10): Chanthaburi farmers placed a higher value on HAB and BEEKP than Chiang Mai farmers, while comparatively caring less about NB_DEC. The regional dummy thus affected the part-worth means individually, rather than acting proportionally over all attributes, i.e., we keep *H2*.*3*. A non-significant effect of CHB on scale implies that farmers in Chanthaburi and Chiang Mai have equal *E*[*λ*_*i*_] and therefore, on average, value conservation policy attributes in similar proportions to *ε*_*ij*_. We thus reject *H3*.*2*. On the other hand, such a proportion is random for Chiang Mai farmers (i.e., individual-specific *λ*_*i*_, given the significant *τ* estimate of the preferred model M5), whereas Chanthaburi farmers exhibit constant *λ* (cf. preferred Chanthaburi model M6), i.e., we keep *H1*.*2* for Chiang Mai, yet reject it for Chanthaburi.

As a consequence of discarding a regional effect on scale (according to the preferred model M10), we can also rule out any significant effect on scale from differences in the underlying experiments, which indeed were designed differently for both regions. Moreover, although an effect on scale could neither be rejected for BEE_FARM in Chiang Mai, nor for MALE in Chanthaburi (correspondingly Tables 3 and 4, and hypotheses *H3*.*3* and *H3*.*4*), the addition of these parameters did not contribute to improvements in model performance.

The comparatively lower WTP of Chanthaburi farmers to avoid a 50% decline in native bee populations (M15) may result from the locally widespread adoption of traditional beekeeping (predominantly stingless bee spp.) that has endowed their farms with crop pollination independence, which otherwise is primarily mediated by wild bees. In contrast, traditional beekeeping in Chiang Mai mostly relies on *A*. *cerana* bees that are baited into man-made hives from which they eventually abscond back into their natural habitat (i.e., unlike stingless bees, which, once captured, can be kept indefinitely in box hives). At any rate, being a beekeeper plays an important role in forming preferences for the implementation of a native bee husbandry measure. This is also true for Chanthaburi, if we take into account that most farmers there are also beekeepers and that in an unreported model (cf. Section 3.3), the WTP for BEEKP only came out as significant for those individuals who keep bees. This leads us to keep *H2*.*1*. However, that model is less preferred than M11 probably because most farmers (60%) in Chanthaburi are also beekeepers. Furthermore, the notion of a possible native bee-pollination shortage (POLL_DEC) had a positive, yet ambiguous effect on Chanthaburi’s farmers’ preference for BEEKP (i.e., the beekeeping measure), which may result from POLL_DEC being confounded with BEEKEEPER (i.e., being a beekeeper) in this province. No confident decision could thus be taken regarding *H2*.*2*.

By all means, one should be careful with the interpretation of the WTP estimates for the percentage changes in the population of native bees, as these considerably exceed the highest implementation cost presented in the choice cards (i.e., THB750). One should also be wary not to infer about the entire population of targeted Chanthaburi farmers, considering that it was not possible to survey this population in a representative fashion.

We would like to close this section by situating this study’s GMXL application in the context of following recent discussion: According to Hess and Train [63], scale heterogeneity is a form of correlation in the part-worths that cannot be separately identified from other sources of heterogeneity. Furthermore, Hess and Rose [57] already warned that models such as the GMXL, with which a number of authors try to disentangle scale heterogeneity from taste heterogeneity, maintain the scale/part-worth confounding and that the gain in model fit from those specifications results from allowing more flexible distributions.

In line with their criticisms, these authors suggest that a RPL specification is sufficient for capturing all sources of correlation, including scale heterogeneity [57, 63]. In fact, Hess and Train [63] argue that GMXL is a restricted form of RPL, unless the part-worths in the latter are assumed to be uncorrelated, in which case they consider the embedding is reversed. They further acknowledge that, if correlation is allowed in a full vector of random part-worths, the GMXL can accommodate scale heterogeneity. Nonetheless, they maintain that modelling scale imposes a restriction relative to RPL, arguing that in doing so, the covariance matrix is being captured by a single (scale) parameter, which draws on the argument that the heterogeneity from scale and from taste cannot be disentangled [63].

Indeed, the GMXL models we fitted (see Tables 3 and 4) do not separately identify scale heterogeneity. Instead, the specification proposed by Fiebig et al. [44] assumes a functional form for the distribution of the scale parameter, such that its expected value equals unity. Thereby, one identifies the parameters that describe such distribution as the coefficients to be estimated, instead of estimating the scale parameter itself. Furthermore, the special case of GMXL that we applied (i.e., GMXL I, cf. Eq 4 and setting *γ* = 1) assigns heterogeneity to taste (through *η*_*i*_, which captures independent unobserved deviations from the mean part-worth vector), separately from the heterogeneity that enters the model as correlation in the part-worth vector [*λ*_*i*_(*β* + Δ*z*_*i*_)], i.e., through the random scale parameter *λ*_*i*_. Having additionally allowed all part-worths to be correlated, our GMXL model specifications produced parameter estimates that separately described the distributions assumed for the part-worth vector (i.e., multivariate normal) and for the scale parameter (i.e., lognormal with *E*[*λ*_*i*_] = 1). The question nevertheless remains as to whether the improved goodness of fit in several of the estimated GMXL models (according to e.g., AIC and BIC) resulted from assuming a mixture of distributions that is more flexible than that assumed for the RPL models (see Tables 3 and 4).

As for the WTP space estimation (see Eq 7), Hess and Train [63] affirm that such models allow for all sources of correlation as long as the price effect enters linearly the underlying utility specification, which is the case in our WTP analyses (see Table 5).

## 5. Conclusions

From the above analyses, we can dismiss the null hypotheses of homogeneous choice behavior between and within Chiang Mai and Chanthaburi farmers regarding alternative native bee conservation policies. We further conclude with confidence that such heterogeneous choices can be partly explained by the influence of observed farmer-specific characteristics on their preference for the single policy-constituting attributes and on the variability with which unobserved factors (i.e., which were not captured in the DCE) contribute to their choices.

Our results suggest that those farmers in Chiang Mai who answered *yes* to the question of whether they believed they had experienced a past bee-mediated pollination deficit derived less utility from the conservation policy measures, relative to other (unobserved) choice decision influences. This result is nevertheless challenged by the lack of evidence for an actual pollinator crisis in Chiang Mai. We therefore suggest that, instead, this question captured the poor understanding of a portion of farmers regarding the agricultural importance of bees, which further led them to base their choice decisions, to a larger degree, on unobserved factors. Similarly, Chiang Mai farmers who keep their own or someone else’s bees on their farm may have incorporated relatively more unobserved factors into their decision process than farmers who do not keep bees. We presume that this effect results from longan farmers of Chiang Mai valuing bees, almost exclusively, for their direct economic benefits: most farmers keep bees that belong to beekeeping entrepreneurs who pay them for the right to forage longan nectar, and the few farmers who keep their own bees probably do it entirely for the hive products.

Engaging in activities that involve bees (which in Chiang Mai is likely to be mainly driven by their direct economic benefits, such as honey, rather than by pollination services) also has a significant effect on the preference for bee husbandry as part of a native bee conservation policy in this region. This finding, initially proposed in the study by Narjes and Lippert [41], is substantiated by the GMXL estimates of the present analyses. Furthermore, this study preserves, to a considerable degree, the orders of magnitude and proportions of the willingness to pay (WTP) estimates for Chiang Mai from Narjes and Lippert [41], thus indicating their robustness.

An interesting result was obtained by modelling the extent to which being a farmer from Chanthaburi influenced the variability of the unobserved contributions to choice decisions: the corresponding estimated positive interaction parameter δ results in a larger scale *λ* of utility and thus in a smaller variance *σ*^2^ of the unobserved residuals for the Chanthaburi subsample. This result in turn suggests that the choices of this group of farmers were more strongly informed by the observed conservation policy attributes than the choices of the Chiang Mai subsample. Nonetheless, perhaps the most important finding of this study is that the WTP for a native bee conservation policy was, in general, significantly higher in Chanthaburi than in Chiang Mai. The fact that the majority of Chanthaburi farmers (~60%) are also beekeepers (which in this region correlates with the notion of a past bee-mediated pollination deficit), makes it difficult to differentiate their value perceptions from those farmers who do not keep bees. Moreover, it suggests that the comparatively higher WTP of Chanthaburi farmers may result from the farmers’ actual need to manage their own crops’ pollination.

## 6. Policy implications

We propose that an actual local pollinator decline may have made Chanthaburi farmers more aware of the importance of native bees for crop pollination, while (paradoxically) making them more independent from the provision of wild pollination services, as they started managing crop pollination with stingless bees to supplement (apparent) pollination deficits. This hypothesis is supported by the comparatively higher WTP of Chanthaburi farmers for the implementation of the native bee husbandry and improved native bee habitat measures, and is further corroborated by the fact that they usually collect stingless bee nests in the surrounding natural habitats (i.e., before being reared in the farms). Furthermore, the comparatively lower WTP of Chanthaburi farmers for the bee-friendly pest management and to avoid native (wild) bee population declines corroborates their independence from the provision of wild pollination services, especially considering that the shorter flight range of stingless bees protects them from pesticides exposure. In view of this, we further submit that waiting for a pollinator crisis to set in, before a conservation policy has been implemented, may steer farmers’ preferences toward corrective efforts that are likely to be selective in favor of a few manageable bee species.

There are only a few species of manageable honeybees and stingless bees in Thailand [6]. Relying solely on bee husbandry for their conservation may pose the risk of neglecting the rest of the native pollinator fauna, which already contribute substantially to crop pollination and may serve as important insurance in the event of managed bee shortages [64, 65]. Instead, the implementation of a preventive policy would be likelier to conserve the broader pollinator fauna, including those wild species that are rarely observed near crops. A native bee conservation policy should thus integrate all three IPI-POA recommended adaptive management strategies (presented in this study) and further raise awareness of the importance of pollinators among the general public and special interest groups through the dissemination of high quality and easy-to-understand information [40]. Such a conservation policy should be seen as an investment, considering that the calculated costs of implementing the proposed conservation strategies would only amount to a fraction of the potential production losses that a bee-pollination deficit could entail [41]. Moreover, by taking into account the preferences of pollination-dependent crop farmers with regards to the conservation strategies constituting such a policy (as estimated in this study), one could make ex-ante policy recommendations based on which strategies can be expected to engage more efforts and resources from the targeted farming communities and which ones will require a greater government intervention.

On average, the three proposed conservation measures were valued positively, yet all models coincide in their significantly wide-ranging standard deviations. Although expected (and partly explained in this study), this result poses a challenge for the implementation of these measures; to increase these policies’ chances of success, policy makers could gain further insights from qualitative analyses that try to explain such part-worth heterogeneity.
